# Predictors of calcification distribution in severe tricuspid aortic valve stenosis

**DOI:** 10.1007/s10554-021-02248-6

**Published:** 2021-04-20

**Authors:** Kerstin Piayda, Lisa Dannenberg, Saif Zako, Oliver Maier, Georg Bosbach, Amin Polzin, Shazia Afzal, Christian Jung, Ralf Westenfeld, Malte Kelm, Tobias Zeus, Verena Veulemans

**Affiliations:** 1 Division of Cardiology, Pulmonology and Vascular Medicine, Medical Faculty, Heinrich Heine University, Moorenstr. 5, 40225 Düsseldorf, Germany; 2 CARID (Cardiovascular Research Institute Düsseldorf), Moorenstr. 5, 40225 Düsseldorf, Germany

**Keywords:** Severe aortic stenosis, TAVR, Aortic valve calcification distribution, Multi‐sliced computed tomography analysis, Leaflet calcification

## Abstract

We investigated aortic valve calcification (AVC) distribution and predictors for leaflet calcification patterns in patients with severe tricuspid aortic valve stenosis undergoing transcatheter aortic valve replacement (TAVR). Patients undergoing routine multi-sliced computed tomography (MSCT) for procedural planning were enrolled. MSCT data were transferred to a dedicated workstation for evaluation (3mensio Structural Heart™, Pie Medical Imaging BV, Maastricht, The Netherlands) and analyzed. Participants were separated into asymmetrical (AC) and symmetrical (SC) leaflet calcification and potential predictors for calcification distribution were identified with univariate and multivariate regression analysis. 567 Participants with severe tricuspid AS were divided into asymmetrical (AC, n = 443; 78.1%) and symmetrical (SC, n = 124; 21.9%) AVC. In AC, the non-coronary cusp was the most calcified cusp (n = 238; 57.7%). SC is more common in females (AC/SC: 49.2% vs. 67.7%; p < 0.0001). AVC was more severe in patients with AC, who also have larger aortic root dimensions. Multivariate analysis depicted, inter alia, left ventricular outflow tract (LVOT) calcification < 25 Agatston units (OR 1.81 [1.09–3.00], p = 0.021), a mean pressure gradient < 36 mmHg (OR 1.77 [1.03–3.05], p = 0.039), and an annulo-apical angle > 67° (OR 1.68 [1.00–2.80], p = 0.049) as predictors for SC, although with only moderate predictive value. Data from this retrospective analysis indicate that SC occurs more frequently in females. The cumulative leaflet calcification burden is higher in patients with AC, who also present with larger aortic root dimensions. The predictive value for prominent calcification of different aortic valve cusps in AC patients was only low to moderate.

**Trial registration number**: NCT01805739.

**Supplementary Information:**

The online version contains supplementary material available at 10.1007/s10554-021-02248-6.

## Introduction

Aortic valve stenosis (AS) is the most common valvular heart disease in western countries [1]. The extent of aortic valve calcification (AVC), measured by multislice computed tomography (MSCT), correlates well with the degree of AS severity [2, 3], and is an integral part of current guideline recommendations for the management of patients with valvular heart disease [4]. While sex- and severity-related distribution of aortic valve calcification are well-researched [5, 6], very little is known about predictors for (a)symmetrical leaflet calcification, which can be frequently observed in daily clinical practice.

Therefore, we performed an in-depth analysis of calcium distribution patterns and favoring, underlying conditions in patients with severe, tricuspid AS.

## Methods

### Study population

We retrospectively enrolled 567 patients with severe tricuspid AS, who underwent routine pre-procedural planning for transcatheter aortic valve replacement (TAVR) at the Heart Center Düsseldorf. Patients with prior aortic valve replacement and bicuspid aortic valve were excluded to guarantee comparability between groups. AS severity was defined according to the current European guideline recommendations for the management of patients with valvular heart disease [6]. Patients were separated into an asymmetrical (AC) and a symmetrical (SC) leaflet calcification pattern. In case of AC, participants were further divided by the dominant calcified cusp (non-coronary; NCC, left coronary; LCC and right coronary cusp; RCC). Asymmetrical leaflet calcification was defined as a difference of > 150 Agatston Units (AU) in-between the three leaflets, which is also visibly subsumable.

All patients provided written informed consent for the use of clinical, procedural, and follow-up data for research. The study procedures are in accordance with the Declaration of Helsinki. All necessary ethical oversight was secured: the Local Ethics Committee approved the study protocol (4080) and the study is registered at clinical trials (NCT01805739).

### Statistical analysis

Continuous data are described by means with standard deviation, median with upper and lower 95% confidence interval (CI) or interquartile ranges (IQR). Categorical variables are expressed by frequencies and percentages of a whole. Sex-related differences are analysed with the 2-sided Student’s t test for continuous variables and the chi-square test (Fisher’s exact test) for categorical variables. Univariate and multivariate analysis are used to identify dependent and independent predictors for asymmetric and symmetric calcification distribution. Others calcification distribution patterns were excluded due to the small sample size (NCC/LCC; NCC/RCC; LCC/RCC) Only co-variates with a p-value below 0.1 in the univariate analysis qualified for multivariate binary logistic regression. Receiver-operating-characteristic (ROC) curves are described as c-indices (area-under-the-curve) with 95% CI. The data analysis was performed with SPSS (version 22.0, SPSS, Inc., Chicago, IL) and GraphPad Prism (version 6.0, GraphPad Software, San Diego, CA). All statistical tests were 2-sided, and a p-value < 0.05 was considered statistically significant.

### MSCT imaging acquisition protocol and three‐dimensional (3D) reconstruction

Pre-procedural cardiac MSCT was routinely performed as native and contrast-enhanced multi-slice CT in all patients. CT data were obtained using a 128-slice, single source CT-scanner with temporal resolution of 150 ms and a collimation of 128 × 0.6 mm (“SOMATOM Definition AS+”, Siemens Healthcare, Forchheim, Germany). Images were taken in accordance with TAVR-related standardized recommendations for CT image acquisition [7]. They were transferred to a dedicated workstation for evaluation (3mensio Structural Heart™, Pie Medical Imaging BV, Maastricht, The Netherlands) and reconstructed in the coronal, sagittal and axial planes. After identification of the virtual annular plane, three hinge points were set on the axial plane, and 3D volume-rendered reconstruction initiated. We assessed the calcium amount of the aortic valve and the upper left ventricular outflow tract (LVOT) within 1 cm below the annulus. The calcification was re-calculated in Agatston Units (AU) for the overall- and separated leaflet calcium burden assessment. Calcium originating from extra-valvular structures, such as the mitral valve annulus, the ascending aorta, and the coronary arteries was cropped.

## Results

### Baseline characteristics

567 Patients were divided either into an asymmetrical (AC, n = 443; 78.1%) or a symmetrical (SC, n = 124; 21.9%) leaflet calcification pattern. In the AC group, the NCC was the most calcified cusp (n = 238; 57.7%), followed by the RCC (n = 72; 12.7%) and the LCC (n = 58; 10.2%). Dominating NCC/RCC calcification was observed in 6.3% (n = 36) of patients, followed by a pronounced NCC/LCC calcification in 4.8% (n = 27), and prominent LCC/RCC calcification pattern in 2.1% (n = 12) of cases.

SC was more common in females (AC/SC: 49.2% vs. 67.7%; p < 0.0001) and was associated with various baseline characteristics such as immunosuppression (AC/SC: 2.7% vs. 8.7%; p = 0.014) and chronic obstructive pulmonary disease (COPD) (AC/SC: 28.4% vs. 45.2%; p = 0.001). Other comorbidities, rheological factors, and hemodynamic values were comparable between groups.

In patients with AC, a lower wedge pressure (AC/SC PCWP: 16.7 ± 8.1 mmHg vs. 20.5 ± 5.1 mmHg; p = 0.005), a smaller aortic valve area (AC/SC AVA: 0.7 ± 0.2 cm^2^ vs. 0.8 ± 0.2 cm^2^; p = 0.009), and higher transvalvular gradients (AC/SC mean pressure gradient: 62.2 ± 23.2 mmHg vs. 54.2 ± 23.2 mmHg; p = 0.001) were observed. Overall, patients with AC have an overall increased calcification burden (AC/SC: 2208 AU [1188–2906] vs. 1143 [495–1641]; p < 0.001) an larger aortic root dimensions as compared to SC patients. Further baseline information are displayed in Table 1.

**Table 1:** Patients’ clinical and functional characteristics

	Overall (n = 567; 100%)	Asymmetrical (AC) (n = 443; 78.1%)	Symmetrical (SC) (n = 124; 21.9%)	p-value
Calcium distribution				
Non-coronary cusp	238 (42.0)	238 (42.0)	**–**	**–**
Right coronary cusp	72 (12.7)	72 (12.7)	**–**	**–**
Left coronary cusp	58 (10.2)	58 (10.2)	**–**	**–**
Non-coronary cusp = Right coronary cusp	36 (6.3)	36 (6.3)	**–**	**–**
Non-coronary cusp = Left coronary cusp	27 (4.8)	27 (4.8)	**–**	**–**
Left coronary cusp = Right coronary cusp	12 (2.1)	12 (2.1)	**–**	**–**
Clinical data				
Age (years)	81.8 ± 5.7	81.8 ± 5.6	81.7 ± 5.8	0.804
Female	302 (53.3)	218 (49.2)	84 (67.8)	< 0.0001*
Body mass index	26.7 ± 4.7	26.6 ± 4.5	27.1 ± 5.3	0.262
Malignancy	14 (2.5)	10 (2.3)	4 (3.2)	0.519
Coronary artery disease	408 (72.2)	323 (73.2)	85 (68.5)	0.740
Previous percutaneous coronary intervention	225 (39.7)	183 (41.3)	42 (33.9)	0.063
Previous coronary artery bypass grafting	64 (11.3)	53 (12.0)	11 (8.9)	0.422
Arterial hypertension	517 (91.2)	404 (91.1)	113 (91.2)	1.000
Pulmonary hypertension	350 (61.8)	268 (60.6)	82 (66.1)	0.296
Diabetes mellitus	173 (30.5)	128 (28.9)	45 (36.3)	0.158
Insulin-dependent	72 (12.7)	50 (11.3)	22 (17.7)	0.067
Immunosuppression	22 (3.9)	12 (2.7)	10 (8.1)	*0.014*
Smoker	95 (16.8)	79 (17.8)	16 (12.9)	0.222
Previous pacemaker	77 (13.6)	57 (12.9)	20 (16.1)	0.374
Chronic obstructive pulmonary disease	182 (32.1)	126 (28.4)	56 (45.2)	0.001*
Atrial fibrillation	228 (40.2)	180 (40.6)	48 (38.7)	0.756
Porcelain aorta	51 (9.0)	40 (9.0)	11 (8.9)	1.000
Medication				
Oral anticoagulation	237 (41.9)	182 (41.2)	55 (44.4)	0.538
Statin	353 (62.4)	280 (63.3)	73 (58.9)	0.402
Rheology				
Creatinine (mg/dl)	1.3 ± 0.9	1.3 ± 0.8	1.3 ± 1.1	0.424
Glomerular filtration rate (ml/min)	55.5 ± 20.0	56.0 ± 20.2	53.8 ± 19.6	0.274
Hemoglobin (g/dl)	12.3 ± 5.0	12.4 ± 5.6	12.1 ± 1.6	0.532
Functional data				
Log EuroSCORE (%)	25.2 ± 14.5	25.3 ± 14.9	24.8 ± 13.2	0.735
Cardiac index (l/min/m^2)^	2.3 ± 0.6	2.4 ± 0.6	2.3 ± 0.5	0.160
Severely reduced LVF (< 35%)	44 (7.8)	33 (7.4)	11 (8.9)	0.789
Aortic regurgitation ≥ II°	99 (17.5)	76 (17.8)	23 (18.7)	0.791
Mitral stenosis ≥ II°	38 (6.8)	29 (6.6)	28 (22.6)	0.839
Mitral regurgitation ≥ II°	126 (22.2)	93 (20.9)	33 (26.6)	0.222
Tricuspid regurgitation ≥ II°	100 (17.7)	76 (17.1)	24 (19.3)	0.595
Systolic pulmonary artery pressure (mmHg)	43.1 ± 15.0	42.6 ± 14.9	44.8 ± 15.0	0.238
PCWP (mmHg)	17.6 ± 9.3	16.7 ± 8.1	20.5 ± 12.1	0.005*
PVR (dynes/cm^5^)	225.5 ± 205.4	225.2 ± 217.2	226.5 ± 159.4	0.964
SVR (dynes/cm^5^)	1821 ± 670.4	1798 ± 687.7	1894 ± 609.6	0.281
LVEDP (mmHg)	21.6 ± 8.8	21.6 ± 8.3	21.6 ± 10.3	0.988
Aortic valve area (cm^2)^	0.8 ± 0.2	0.7 ± 0.2	0.8 ± 0.2	0.009*
Maximum pressure gradient (mmHg)	60.5 ± 23.4	62.2 ± 23.2	54.2 ± 23.2	0.001*
Mean pressure gradient (mmHg)	37.3 ± 15.3	38.3 ± 15.2	33.9 ± 15.6	0.006*
Multi-sliced computed tomography data				
Aortic valve calcification (AU)	1976 [952–2658]	2208 [1188–2906]	1143 [495–1641]	< 0.0001*
Non-coronary cusp (AU)	829 [324–1175]	964 [474–1283]	352 [139–466]	< 0.0001*
Left coronary cusp (AU)	543 [216–745]	599 [260–824]	344 [133–491]	< 0.0001*
Right coronary cusp (AU)	583 [227–763]	655 [270–843]	326 [115–432]	< 0.0001*
Left ventricular outflow tract (AU)	188 [0–186]	182 [0–188]	209 [1**–**120]	0.481
Annulus area (cm^2)^	4.7 ± 1.0	4.8 ± 1.0	4.5 ± 1.0	0.001*
Perimeter (mm)	77.0 ± 8.4	77.6 ± 8.6	74.8 ± 7.5	0.001*
Annulus ellipticity	1.2 ± 0.1	1.2 ± 0.1	1.2 ± 0.1	0.121
Left ventricular outflow tract mean (mm)	23.6 ± 2.8	23.8 ± 2.7	22.9 ± 2.8	0.001*
Left ventricular outflow tract ellipticity	1.4 ± 0.2	1.4 ± 0.2	1.4 ± 0.2	0.030*
Sinotubular junction mean (mm)	29.1 ± 3.3	29.3 ± 6.4	28.3 ± 3.2	0.105
AOA mean (mm)	33.4 ± 3.5	33.4 ± 3.5	33.4 ± 3.6	0.886
Sinus of Valsalva diameter (mm)	35.1 ± 9.3	35.3 ± 9.3	34.2 ± 9.3	0.259
Sinus of Valsalva radius NCC (mm)	17.9 ± 5.0	18.1 ± 5.0	18.4 ± 4.9	0.167
Sinus of Valsalva radius LCC (mm)	17.8 ± 4.9	17.9 ± 4.9	17.4 ± 4.7	0.346
Sinus of Valsalva radius RCC (mm)	17.1 ± 4.7	17.2 ± 4.7	16.6 ± 4.7	0.186
Right coronary artery distance (mm)	15.0 ± 3.1	15.1 ± 3.1	14.7 ± 3.1	0.197
Left coronary artery distance (mm)	12.3 ± 2.7	12.4 ± 2.7	11.8 ± 2.6	0.029*
Right coronary cusp length (mm)	10.5 ± 2.1	10.6 ± 2.1	10.2 ± 1.8	0.037*
Left coronary cusp length (mm)	11.0 ± 2.0	11.1 ± 2.0	10.5 ± 2.0	0.005*
Ratio Right coronary artery/Right coronary cusp	1.5 ± 0.4	1.5 ± 0.4	1.5 ± 0.3	0.784
Ratio Left coronary artery/Left coronary cusp	1.2 ± 0.3	1.1 ± 0.3	1.1 ± 0.2	0.812
Aortic root angulation (°)	48.9 ± 10.7	49.3 ± 10.3	47.7 ± 11.8	0.191
Annulo-apical angulation (°)	67.5 ± 13.7	67.1 ± 13.2	69.1 ± 15.3	0.208

*Significant level p < 0.05 (bold, italics)

Values are mean ± SD, mean ± 25th and 75th percentile or n (%)

*AU* Agatston units, *BMI* body mass index, *CABG* coronary artery bypass graft, *CAD* coronary artery disease, *CI* cardiac index, *COPD* chronic obstructive pulmonary disease, *CVD* cerebrovascular disease, *dPmean*/*max* mean/max. transvalvular gradient, *LCC* Left coronary cusp, *LVEDP* Left ventricular enddiastolic pressure, *LVEF* Left ventricular ejection fraction, *LAO* left anterior oblique, *LVOT* Left ventricular outflow tract, *NCC* Non-coronary cusp, *PCI* percutaneous coronary intervention, *PHT* pulmonary hypertension, *RCC* Right coronary cusp, *SOV* Sinus of Valsalva, *STJ* Sinotubular junction

### Univariate and multivariate predictors for calcification distribution patterns

#### Symmetrical calcification

Multivariate analysis depicted the presence of COPD (OR 2.15 [1.26–3.65], p = 0.005), an LVOT calcification < 25AU (OR 1.81 [1.09–3.00], p = 0.021), a mean gradient below 36 mmHg (OR 1.77 [1.03–3.05], p = 0.039), and an annulo-apical angulation above 67° (OR 1.68 [1.00–2.80], p = 0.049) as predictive for a SC pattern. However, c-statistics—even when combined—only showed a moderate correlation (Table 2).

**Table 2:** Discrimination performance (ROC and AUC statistics)

Calcification site	Parameters	AUC	p-value	Lower 95% CI	Upper 95% CI
Symmetrical	Chronic obstructive pulmonary disease	0.58	0.025*	0.51	0.65
	Left ventricular outflow tract < 25 AU	0.60	0.005*	0.53	0.66
	Mean pressure gradient < 36 mmHg	0.59	0.009*	0.52	0.66
	Annulo-apical angulation > 67°	0.56	0.089	0.49	0.63
	Combined AUC	0.68	< 0.0001*	0.62	0.74
Non-coronary cusp	Previous coronary artery bypass grafting	0.53	0.233	0.48	0.58
	No porcelain aorta	0.53	0.342	0.48	0.58
	Combined AUC	0.55	0.05*	0.50	0.60
Left coronary cusp	–	–	–	–	–
RCC	No permanent pacemaker	0.56	0.157	0.48	0.63
	Annulus ellipticity < 1.22	0.60	0.012*	0.52	0.68
	Ratio right coronary artery/right coronary cusp > 1.43	0.58	0.040*	0.51	0.66
	Annulo-apical angulation < 67°	0.57	0.099	0.49	0.64
	Combined AUC	0.68	< 0.0001*	0.61	0.75

*Significant level p < 0.05 (bold, italics)

Values are mean ± SD, mean ± 25th and 75th percentile or n (%)

*AU* Agatston units, *CABG* coronary artery bypass graft, *COPD* chronic obstructive pulmonary disease, *LCC* Left coronary cusp, *LVOT* Left ventricular outflow tract, *NCC* Non-coronary cusp, *PM* Pacemaker, *RCA* Right coronary artery, *RCC* Right coronary cusp, *SOV* Sinus of Valsalva, *STJ* Sinotubular junction

#### Asymmetric calcification with dominating NCC calcification

Univariate analysis depicted the presence of malignancy, male gender, the absence of a porcelain aorta, previous coronary artery bypass grafting, and a larger aortic anatomy as possible influencing factors for dominant NCC calcification. Multivariate analysis identified the absence of a porcelain aorta (OR 2.03 [1.07–3.86], p = 0.031) and previous coronary artery bypass grafting (OR 1.95 [1.14–3.32], p = 0.0014) as independent predictors for a pronounced NCC-calcification. However, c-statistics—even combined—remained only in a poor range (Table 2).

#### Asymmetric calcification with dominating LCC calcification

Prominent LCC-calcification was linked to a porcelain aorta, a smaller aortic valve area, higher pressure gradients and pronounced LVOT calcification. Multivariate analysis offered no independent predictor for dominating LCC-calcification in patients with AC pattern.

#### Asymmetric calcification with dominating RCC calcification

Concerning RCC calcification univariate analysis identified, inter alia, the absence of a permanent pacemaker (PPM) at baseline, an annulus ellipticity index < 1.22, larger dimension of the sinus of Valsalva, a greater RCA-to-RCC leaflet ratio (RCA/RCC > 1.43) and an annulo-apical angulation < 67° as potential predictors.

**Fig. 1:**
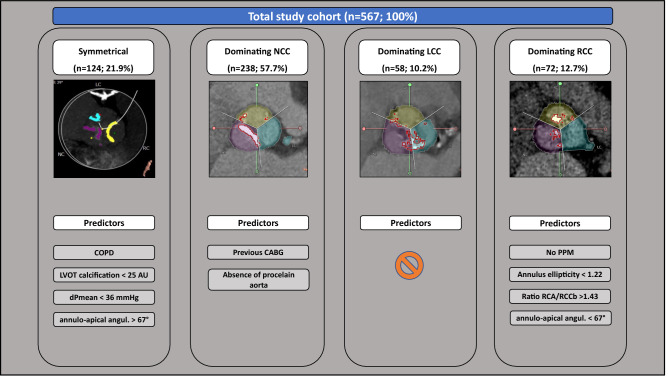
Calcification distribution and independent predictors. The amount of calcium of the aortic valve and surrounding structures were assessed and re-calculated in Agatston Units (AU) for overall- and separated leaflet calcium burden. Symmetrical and asymmetrical leaflet calcification with the dominant calcified cusp (non coronary cusp-magenta; left coronary cusp-blue; right coronary cusp-yellow) are displayed with independent predictors—identified by multivariate analysis. *CABG* coronary artery bypass grafting, *COPD* chronic obstructive pulmonary disease, *dPmean* mean pressure gradient, *PPM* permanent pacemaker, *Ratio RCA*/*RCCb* ratio of the right coronary artery to the base of the right coronary cusp

In a multivariate analysis the absence of PPM at baseline (OR 6.01 [1.40–25.78], p = 0.016), an annulus ellipticity < 1.22 (OR 2.78 [1.55–4.97], p = 0.001), an RCA/RCC leaflet ratio > 1.43 (OR 2.04 [1.15–3.65], p = 0.016), and an annulo-apical angulation < 67° (OR 1.98 [1.11–3.55], p = 0.022) proved to be independent predictive factors for prominent RCC calcification. c-Statistics remained only in a moderate range (Table 2). A graphical illustration of the read-out is given in Fig. 1. Detailed results of uni- und multivariate regression analysis can be found in the Supplement (Table 3).

## Discussion

To our knowledge, this is the first study with a systematic and in-depth analysis of AVC distribution, which includes comorbidities, hemodynamic parameters, and anatomical pre-dispositions of patients with severe tricuspid AS undergoing TAVR.

Our retrospective study revealed that:

- A symmetrical calcification pattern is more frequently observed in females who have narrow aortic root dimensions.
- Cumulative leaflet calcification is higher in patients with asymmetrical leaflet calcification patterns.
- Independent predictors for prominent calcification of single aortic valve cusps vary widely and have only poor to moderate predictive value:
- Independent predictors for symmetrical leaflet calcification are COPD, a less pronounced LVOT calcification, lower mean pressure gradients, and a more horizontal aorta.
- Independent predictors for pronounced NCC calcification are previous CABG and the absence of a porcelain aorta.
- Independent predictors for a prominent RCC calcification are the absence of a PPM, a less elliptical annulus index, a higher RCA/RCC ratio, and a less horizontal aorta..

### Asymmetrical vs. symmetrical AVC distribution

Koshkelashvili et al. retrospectively analyzed 318 non-contrast axial chest CT scans of subjects aged over 65 years in an all-comers cohort. They could also show that the LCC was frequently the most calcified cusp [8]. The study was not performed in patients solely presenting with AS but provided important information on race-related differences in early calcification patterns. In our study, race-differences were not considered to be relevant since over 99% of the study population were Caucasian. Overall, the number of studies concerning AVC patterns is limited since AVC is pre-dominantly examined in total and semi-quantitatively during pre-procedural planning for TAVR [9, 10]. It has already been shown that AVC distribution is linked to important clinical endpoints such as the occurrence of conduction disturbances, the risk of annular rupture, coronary occlusion or paravalvular leakage [11–14]. Especially asymmetric calcification, possibly leading to increased cardial forces on the NCC and RCC and adjacent structures such as the intramembranous septum, and pronounced LCC calcification are relevant variables for a permanent pacemaker implantation post TAVR [15, 16].

Current studies do not provide insight in pre-dispositioning factors, which are linked to the presented calcification pattern in patients with severe tricuspid AS. Furthermore, fibrosis is an essential factor in degenerative AS, leading to higher-grade stenosis under non-severe AVC-thresholds, especially in women. Sex-related differences in the progression and clinical phenotypes of aortic valve stenosis are well researched. High AVC is more likely in men and shows a strong correlation with the severity of AS in both sex [6]. Woman have a slower progression rate of AS but lower calcium levels are already sufficient to create the same hemodynamic impairment as in men with higher AVC load [17]. This might be explained by the fact that women are more prone to higher levels of valvular fibrosis and dense connective tissue at the same degree of hemodynamic relevant aortic stenosis severity as compared to their male counterparts [18]. Since the SC group primarily consists of women, the lower aortic valve gradients and the lower overall AVC load may be explained by the aforementioned facts.

Shear forces, aortic root entrance angles, and flow-patterns may also play an important role in AVC distribution and should be further analyzed. A horizontal aorta, the extreme form of increased aortic root angulation and the annulo-apical entrance angles, is known to limit maneuverability of self-expandable devices [19, 20] but also means a potential shear force trigger, probably leading to flow-dependent calcification. However, flow is pre-dominantly linked to myocardial function. Surprisingly, neither cardiac output, higher-grade reduced left ventricular ejection fraction or other hemodynamic characteristics in this context took a particular influence on calcification distribution, supposing that calcification may be more significantly linked to aortic flow characteristics.

### Dominant leaflet calcification in patients with AC

Permanent ventricular pacing was shown to be associated with alterations in regional myocardial and coronary perfusion [21] and may, therefore, be linked to altered calcification patterns. Coronary artery disease was no dependent or independent predictor in our analysis, but the distribution of coronary artery disease and the distance of the coronary arteries might have an impact on AVC patterns through altered sinus perfusion and connected ostial calcification areas. Effacement of the sinuses may impair coronary flow and increase the mechanical stress and thus structural degeneration of the aortic valve leaflets [22]. In bicuspid valves, deterioration of aortic blood flow or eccentric flow is well-known to increase radial pressure and shear stress on the aortic wall [23], also causing enhanced dilatation of the aortic root, closing the circle towards aortic entrance angles and a horizontal aorta in tricuspid valves. A sub-analysis of the TAVI-WIN Registry [24] could show that increased calcium volumes of the RCC were an independent predictor for new pacemaker implantation after TAVR, whereas increased calcium volume of the NCC had a protective effect. However, findings are highly contradictive throughout current literature [14, 25]. Pathophysiologically, it remains unclear how different calcification patterns are created: in the initiation phase of disease, endothelial damage allows lipid infiltration and subsequent inflammation. In the propagation phase pro-fibrotic pathways and microcalcification smooth the way for further calcification [26]. Biomechanical studies could link high strain to the formation of calcific noduli and disease progression [27, 28]. Therefore, variable cusp geometries and congestive differences in length may also contribute to different calcification patterns, rather then underlying comorbidities.

### Limitations

This is only a single-center analysis and limited to the retrospective quality of available data, which is also reflected by c-statistics. Furthermore, several important factors, like pressure recovery and ventriculo-arterial impedance, were not analyzed. This study of pre-disposing factors for different calcification patterns lacks translational value and does not influence clinical decision making.

### Future directions

This is the first study trying to determine predictive factors for different types of aortic calcification patterns. Predictive factors vary widely throughout baseline characteristics and only showed poor to moderate correlation. This study may encourage clinicians to perform quantitative calcium assessment measurements in pre-TAVR MSCTs to create further evidence in this field.

## Conclusions

Data from this retrospective analysis indicate that SC occurs more frequently in female patients and narrow aortic root anatomies, whereas cumulative leaflet calcification seems to be higher in AC patterns. Independent predictors for SC are COPD, a less pronounced LVOT calcification, lower mean pressure gradients and a more horizontal aorta. However, the correlation of different baseline characteristics with certain calcification patters were only in poor to moderate range. This is the first study addressing predictors for different calcification patterns, which are known to impact clinical outcomes of patients undergoing TAVR.

## Supplementary Information

Below is the link to the electronic supplementary material.

Supplementary Material 1 DOCX 18 kb

## Data Availability

Data will be made available up on reasonable request by the corresponding author.
