# Supplementary material for: Predictors of calcification distribution in severe tricuspid aortic valve stenosis
Source: Int J Cardiovasc Imaging. 2021 Apr 20;37(9):2791–9. doi: 10.1007/s10554-021-02248-6 (PMC8390394; doi:10.1007/s10554-021-02248-6)
Supplement: Supplementary file 1 — Supplementary Material 1 DOCX 18 kb [file 10554_2021_2248_MOESM1_ESM.docx]

**Appendix**

**Table 3: Univariate and multivariate regression analysis with dominating calcification as the dependent variable**

| **Dominating calcification site** | **Univariate analysis** | | **Multivariate analysis** | |
| --- | --- | --- | --- | --- |
| 1. **Symmetrical calcification** |  |  |  |  |
| Female | 2.17 (1.42-3.30) | *<0.0001** | - | *-* |
| Insulin dependt diabetes mellitus | 1.70 (0.98-2.93) | 0.058 | - | *-* |
| Chronic obstructive pulmonary disease | 2.07 (1.38-3.12) | *<0.0001** | 2.15 (1.26-3.65) | *0.005** |
| Immunosuppression | 3.15 (1.33-7.48) | *0.009** | - | *-* |
| No previous aortic surgery | 2.37 (0.91-6.14) | 0.076 | - | *-* |
| No ASS medication | 1.41 (0.94-2.10) | 0.098 | - | *-* |
| LVOT calcification < 25 AU | 1.59 (1.06-2.38) | *0.025** | 1.81 (1.09-3.00) | *0.021** |
| Aortic valva ares >0.73 cm^2^ | 1.65 (1.10-2.49) | *0.016** | - | *-* |
| Maximum pressure gradient <60 mmHg | 1.73 (1.14-2.64) | *0.010** | - | *-* |
| Mean pressure gradient <36 mmHg | 1.56 (1.03-2.37) | *0.035** | 1.77 (1.03-3.05) | *0.039** |
| Perimeter <76.4 mm | 2.04 (1.35-3.07) | *0.001** | - | *-* |
| Annulus diameter <24 mm | 1.87 (1.25-2.80) | *0.002** | - | *-* |
| Annulus area <4.6 cm^2^ | 1.75 (1.16-2.64) | *0.007** | - | *-* |
| Sinus of valsalve diameter <33 mm | 2.09 (1.38-3.16) | *<0.0001** | - | *-* |
| Sinotubular junction diameter <29 mm | 1.64 (1.09-2.48) | *0.019** | - | *-* |
| Left ventricular outflow tract diameter <24 mm | 2.13 (1.38-3.27) | *0.001** | - | *-* |
| Left ventricular outflow tract ellipticity >1.37 | 1.50 (1.00-2.24) | 0.050 | - | *-* |
| Left coronary cusp length >11mm | 1.91 (1.25-2.91) | *0.003** | - | *-* |
| Annulo-apical angulation >67° | 1.48 (0.93-2.33) | 0.096 | 1.68 (1.00-2.80) | *0.049** |
| 1. **Non-coronary cusp** | **Odds ratio (95-CI)** | **p-value** | **Odds ratio (95-CI)** | **p-value** |
| Malignancy | 2.56 (0.85-7.73) | 0.096 | - | *-* |
| No porcelain aorta | 1.83 (0.98-3.42) | 0.060 | 2.03 (1.07-3.86) | *0.031** |
| Male | 1.41 (1.01-1.97) | *0.045** | - | *-* |
| Previous coronary artery bypass grafting | 1.78 (1.05-3.00) | *0.031** | 1.95 (1.14-3.32) | *0.014** |
| Mitral stenosis >II° | 1.84 (0.89-3.78) | 0.099 | - | *-* |
| Perimeter >76.4 mm | 1.39 (0.99-1.94) | 0.057 | - | *-* |
| Annulus diameter >24 mm | 1.35 (0.96-1.89) | 0.081 | - | *-* |
| Right-coronary cusp length >10 mm | 1.35 (0.96-1.89) | 0.081 | - | *-* |
| 1. **Left coronary cusp** |  |  |  |  |
| Porcelain aorta | 2.04 (0.94-4.54) | 0.072 | - | *-* |
| No chronic obstructive pulmonary disease | 1.73 (0.91-3.29) | 0.097 | - | *-* |
| Left ventricular outflow tract calcification >25 AU | 1.72 (0.99-3.01) | 0.056 | - | *-* |
| Aortic valve area <0.73 cm^2^ | 1.66 (0.95-2.92) | 0.076 | - | *-* |
| Maximum pressure gradient > 60 mmHg | 1.69 (0.96-2.95) | 0.067 | - | *-* |
| Mitral stenosis >II° | 2.55 (1.11-5.86) | *0.028** | - | *-* |
| Annulo-apical angulation < 67° | 1.73 (0.92-3.25) | 0.092 | - | *-* |
| 1. **Right-coronary cusp** |  |  |  |  |
| No previous pacemaker | 2.28 (0.89-5.85) | 0.086 | 6.01 (1.40-25.78) | *0.016** |
| Annulus ellipticity <1.22 | 1.60 (0.97-2.64) | 0.065 | 2.78 (1.55-4.97) | *0.001** |
| Sinus of valsalva >33 mm | 1.56 (0.95-2.58) | 0.081 | - | *-* |
| Ratio right coronary artery/right coronary cusp >1.43 | 1.55 (0.94-2.56) | 0.087 | 2.04 (1.15-3.65) | *0.016** |
| Annulo-apical angulation <67° | 1.72 (0.98-3.00) | 0.058 | 1.98 (1.11-3.55) | *0.022** |
